# Supplementary material for: Passive Daytime Radiative Cooling of Silica Aerogels
Source: Nanomaterials (Basel). 2023 Jan 24;13(3):467. doi: 10.3390/nano13030467 (PMC9919039; doi:10.3390/nano13030467)
Supplement: Supplementary file 1 [file nanomaterials-13-00467-s001.zip › nanomaterials-2150928-supplementary.pdf]

Supporting Information for

Passive Daytime Radiative Cooling of Silica Aerogels

Bingjie Ma[1,2], Yingying Cheng[2], Peiying Hu[2], Dan Fang[3] and Jin Wang[1,2,]*

[1] School of Nano-Tech and Nano-Bionics, University of Science and Technology of China, Hefei 230026, P. R. China;

[2] Key Laboratory of Multifunctional Nanomaterials and Smart Systems, Suzhou Institute of Nano-Tech and Nano-Bionics, Chinese Academy of Sciences, Suzhou 215123, P. R. China

[3] Suzhou Institute of Metrology, Suzhou, 215128, P. R. Chin

*Correspondence: jwang2014@sinano.ac.cn

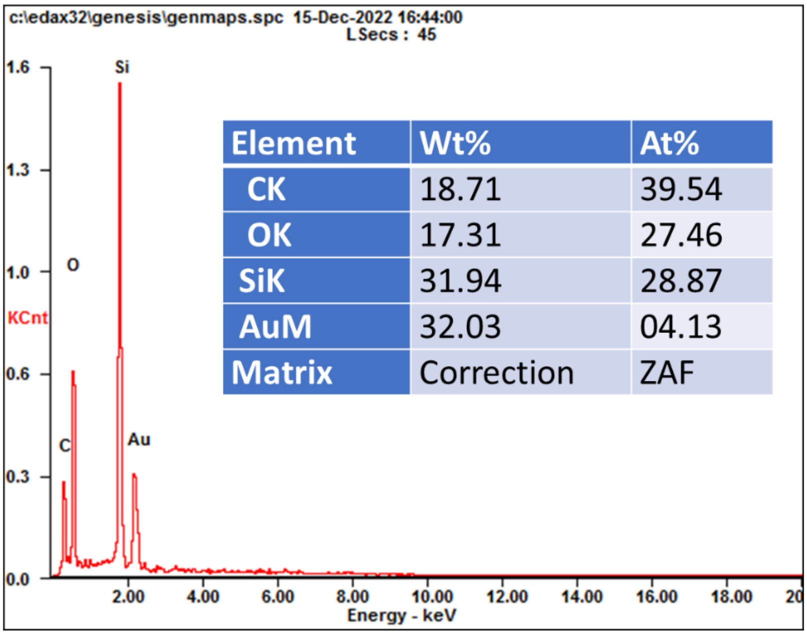

Element	Wt%	At%
CK	18.71	39.54
OK	17.31	27.46
SiK	31.94	28.87
AuM	32.03	04.13
Matrix	Correction	ZAF

Figure S1. Energy-dispersive X-ray spectrometry and elemental contents of the MDA3-2

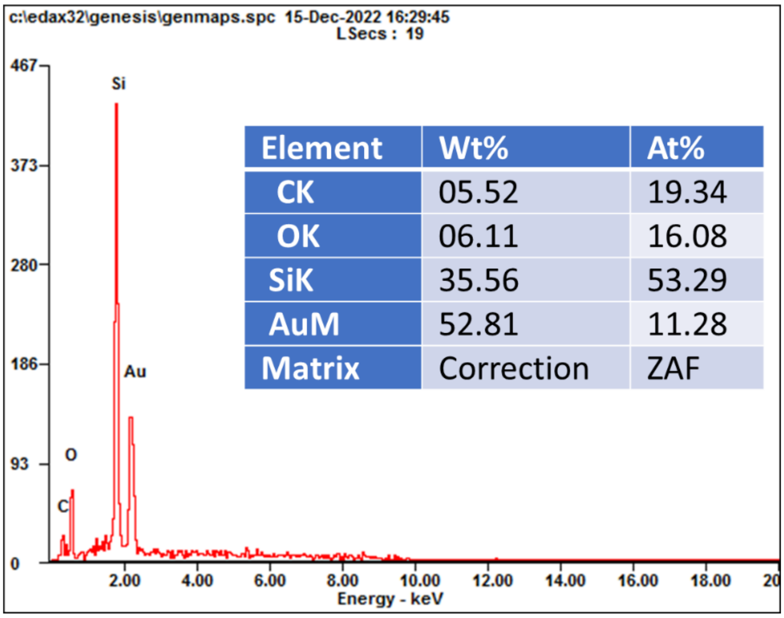

Element	Wt%	At%
CK	05.52	19.34
OK	06.11	16.08
SiK	35.56	53.29
AuM	52.81	11.28
Matrix	Correction	ZAF

Figure S2. Energy-dispersive X-ray spectrometry and elemental contents of the MDA7-3

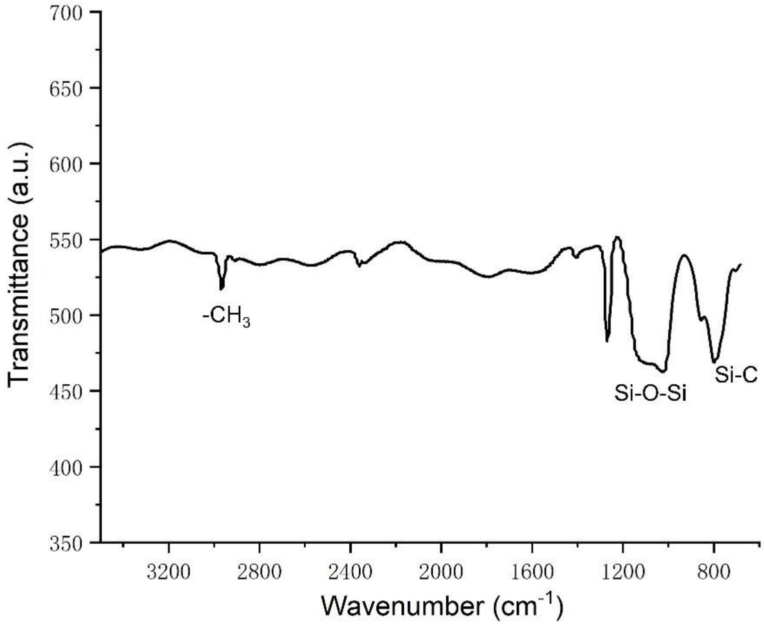

Figure S3. Fourier transform infrared spectrum of the MDA7-3
